# Evaluating single nucleotide polymorphisms in beta - 2 - microglobulin – a theoretical study

**DOI:** 10.3389/fimmu.2025.1622416

**Published:** 2025-09-02

**Authors:** Ammar K. Daoud, Wafa’ A. Alqarqaz, Majduleen M. Al Okor

**Affiliations:** ^1^ Department of Medicine, Faculty of Medicine, Jordan University of Science and Technology, Irbid, Jordan; ^2^ Department of Computer Science, Faculty of Computer and Information Technology, JUST, Irbid, Jordan

**Keywords:** single nucleotide polymorphism, immunoglobulin domain superfamily, beta 2 microglobulin, amino acid substitutions, genetics

## Abstract

**Background:**

In Immunology, many molecules are composed of Immunoglobulin domains. Beta 2 Microglobulin (B2M) is the smallest Immunoglobulin Domain Superfamily member composed of a single domain that is highly conserved in nature in all vertebrates with low rate of Single Nucleotide Polymorphisms (SNP).

**Objective:**

We wanted to mathematically evaluate the effects of the SNP’s-induced Amino Acid (AA) substitutions on the primary structure of the protein.

**Methods:**

A C++ computer program code was written to take the 360 B2M mature DNA nucleotide sequences giving back the corresponding protein sequence of 119 AA. For each nucleotide the corresponding 3 possible SNP’s were generated and the 9 possible modifications per triplet were assessed, taking into consideration critically located AA’s like Cysteine residues involved in disulfide bond formation and the formation of Stop Codons. We used Sneath Score for resemblance of chemical structure to further evaluate the AA Substitutions.

**Results:**

In 22.1% of SNP’s no change in the resulting AA was seen, and in 25.4% of cases a relatively small change was seen with an AA of the same group (Positively Charged, Negatively Charged, Polar, Special and Hydrophobic AA). In 5.3% of the cases, a Stop codon was generated which will lead to an early catastrophic termination of the DNA transcription process. Most cases of SNP’s (47.2%) involve a relatively big change characterized by the substitution by an AA of a totally different chemical group leading to a possibly significant result.

**Conclusions:**

The occurrence of SNP’s in B2M is an important “random” event that can affect the structure of the protein. We attempted to evaluate the effect of SNP’s on the primary structure of B2M and concluded that there is need to improve the computer software programs evaluating the effect of SNP’s and other genetic modifications on the proteins as well as improving the scoring systems evaluating the AA substitutions.

## 1 Introductions

The Immunoglobulin Domain is the fundamental structural unit of many Immune System molecules in nature. It is composed of a polypeptide chain of almost 110 AAs folded into a globular structure held firmly in place by a disulfide bond between the 2 Cysteine AA molecules (1). Each domain is composed of two antiparallel arrays of β strands to form two β-pleated sheets held together by a disulfide bond. This structure is repeated in many molecules in nature like the Antibodies Heavy and Light chains, T Cell Receptor chains and Major Histocompatibility Complex chains and is mainly involved in recognition of other substances. The smallest and isolated Immunoglobulin domain chain is the B2M, best known for being associated with the alpha chain of Class I Histocompatibility Complex Molecules (Classical HLA-A, B, C). These MHC molecules are composed of a long alpha chain of 3 domains that is anchored to the plasma membrane and an associated B2M subunit that is not membrane-bound (2). B2M is highly conserved in nature found on cells from all vertebrate animals (3) and is associated with molecules like the Atypical Class I MHC molecules in human and mice [HLA-G (4), HLA-F, HLA-E (5), CD1 (6), MR1 (7), Qa1 and HFE (8)] mediating many functions still to be discovered. Serum B2M acts as an acute phase reactant (9), and inflammatory marker (10), that is prognostically useful in cases of multiple myeloma (11, 12), Rheumatoid Arthritis (13), Systemic Lupus Erythematosus (14) and Human Immunodeficiency Virus Infection (15). It works as a Host Factor for Vaccinia Virus Infection as identified by genome-wide CRISPR genetic screens (16, 17). Free B2M is present in biological fluids such as urine, spinal fluid, saliva, semen and serum (18), and is found to interact with a molecule (ESAT-6) produced by *Mycobacteria* species thus decreasing their virulence (19).

The genetic makeup determines all the characteristics of an individual by controlling protein structure, but can be modified by many genetic mechanisms like mutations, RNA alternative splicing, epigenetic control and post-translational modifications. Approximately 1.42 million SNP’s occur in less than 1% of the human population at a rate of almost 1 per 1.9 Kb (20). SNP’s usually lead to no or minor modifications in the structure of the final protein, but occasionally lead to severe changes in the structure of the polypeptide chain if occurring in a critical DNA region. Making sense of the effects of the SNP’s depends on the deep and profound understanding of the protein structure at all levels: Primary (AA Sequence), Secondary (Interactions of adjacent AAs), Tertiary (Interactions between distant AAs) and Quaternary (Interactions between Multiple Polypeptide Chains).

In general, the B2M gene is resistant to SNP’s or other types of mutations. In the literature, there were mainly 2 reported diseases resulting from SNP’s in B2M: Familial Hypercatabolic Hypo-proteinemia with Immunodeficiency and Hereditary Systemic Amyloidosis Type 6. In Familial hypercatabolic hypoproteinemia with immunodeficiency due to a defect in the FcRn protein (21), the substitution of Alanine (hydrophobic AA) by Proline (special AA) occurred towards the middle of the polypeptide chain. In hereditary systemic amyloidosis type 6, the substitution of aspartic acid (negatively charged) by asparagine (polar) at position 76 increased the tendency for aggregation and amyloid deposition (22).

In this study, we wanted to mathematically model the effects of all possible SNP’s occurring in the coding region of the B2M gene and evaluate the effects of their resultant AA substitutions on the structure of the Immunoglobulin Domain.

## 2 Methodology

### 2.1 Original DNA, RNA and AA sequence of B2M

The nucleotide sequence of B2M was obtained from the National Center for Biotechnology Information (NCBI) website on 22/10/2024 at 1:30 pm (https://www.ncbi.nlm.nih.gov/nuccore/NM_004048.4) The NCBI reference sequence accession number of B2M is NM_004048.4. The whole gene of B2M on chromosome 15 is composed of 6629 nucleotides and has 4 exons of variable lengths (360, 97, 279 and 28 nucleotides respectively). The mature mRNA is composed of 360 nucleotides from start to Stop codon and encodes a protein of 119 AAs. According to earlier studies, B2M varied in total number of AAs from 96 to 100 but the latest source shows that B2M is made of 119 AAs (2, 18). The following sequence, with the start codon ATG till the stop codon TAA was used in our study.

ATGTCTCGCTCCGTGGCCTTAGCTGTGCTCGCGCTACTCTCTCTTTCTGGCCTGGAGGCTATCCAGCGTACTCCAAAGATTCAGGTTTACTCACGTCATCCAGCAGAGAATGGAAAGTCAAATTTCCTGAATTGCTATGTGTCTGGGTTTCATCCATCCGACATTGAAGTTGACTTACTGAAGAATGGAGAGAGAATTGAAAAAGTGGAGCATTCAGACTTGTCTTTCAGCAAGGACTGGTCTTTCTATCTCTTGTACTACACTGAATTCACCCCCACTGAAAAAGATGAGTATGCCTGCCGTGTGAACCATGTGACTTTGTCACAGCCCAAGATAGTTAAGTGGGATCGAGACATGTAA.

The original sequence of AAs from the source was: -

MSRSVALAVLALLSLSGLEAIQRTPKIQVYSRHPAENGKSNFLNCYVSGFHPSDIEVDLLKNGERIEKVEHSDLSFSKDWSFYLLYYTEFTPTEKDEYACRVNHVTLSQPKIVKWDRDM

### 2.2 C++ code for original DNA sequence analysis

A C++ code was written to read codons of the original sequence of the DNA and generate the corresponding original AA sequence. The original list of AAs was analyzed for the frequency of each AA and its type (Positively Charged or Negatively Charged, Polar, Hydrophobic or Special AAs). The results of the code were exported into an EXCEL sheet for further analysis and graphing.

### 2.3 Analysis of SNP’s on AAs substitutions

For each nucleotide triplet, the 9 corresponding SNPs were generated, and the substituted AA and new AAs as well as their Group were recorded. The original AA and SNP AA pairs were compared, especially for either No Change if the same AA was made or if the change results in the STOP codon type or changes in the groups of AAs (Positively Charged, Negatively Charged, Polar, Hydrophobic or Special AAs). We studied some critically located AAs like the Cysteine where the disulfide bond has to be made (the 2 Cysteine residues are at positions 45 and 100). We used Sneath theoretical score of resemblance between AAs according to the chemical nature and the effects on the physiological properties between them (23). This score ranges from the low score of 5 between Leucine and Isoleucine to the highest score of 43 when the change is between Proline and Glutamic Acid or Proline to Arginine. This score is theoretical, and we used the value of 0 if no change in the AA was found. If the substitution results in a Stop codon, a high score of 100 was used to represent a very high effect. Furthermore, not all AA substitutions are possible by the SNP process.

The C++ Code and the raw data of original AA, SNP AA, Group and Sneath Score comparisons are made available in the **Appendix 1** section.

## 3 Results

### 3.1 Summary of codon representations of AAs

**Figure 1:**
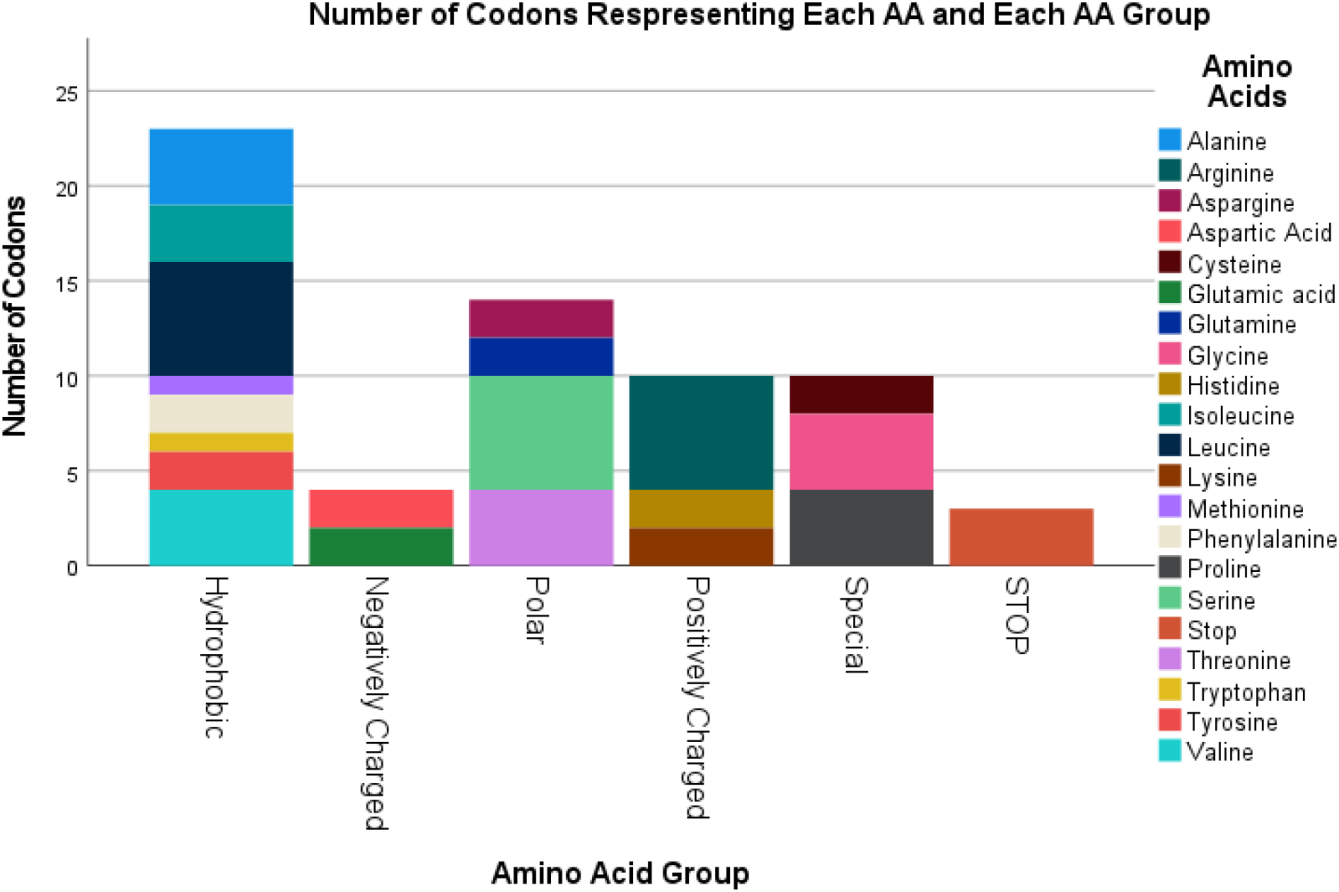
Summary of Codon numbers representing each Amino Acid, according to the Amino Acid Group and Stop codon totaling 64 codons.

Each nucleotide triplet among the 64 possible codons encodes either one natural AA or a Stop signal. The number of codons corresponding to each AA, along with their classification into AA groups is shown in **Figure 1**. 2 AAs are coded by 1 codon (Methionine (representing also the START codon) and Tryptophan), 9 AAs coded by 2 codons (Tyrosine, Phenylalanine, Histidine, Glutamine, Lysine, Glutamic Acid, Aspartic acid, Asparagine and Cysteine). Isoleucine and the STOP codon are coded by 3 codons each. 5 AAs are coded by 4 codons (Valine, Proline, Threonine, Alanine, and Glycine) and 3 AAs are coded by 6 codons (Leucine, Serine and Arginine). As for the type of AA groups, Positively Charged AA are coded by 10 codons, Negatively Charged by 4 codons, Polar by 14 codons, Special by 10 codons, Hydrophobic by 23 codons and STOP by 3 codons.

### 3.2 B2M original AA and AA groups

**Figure 2:**
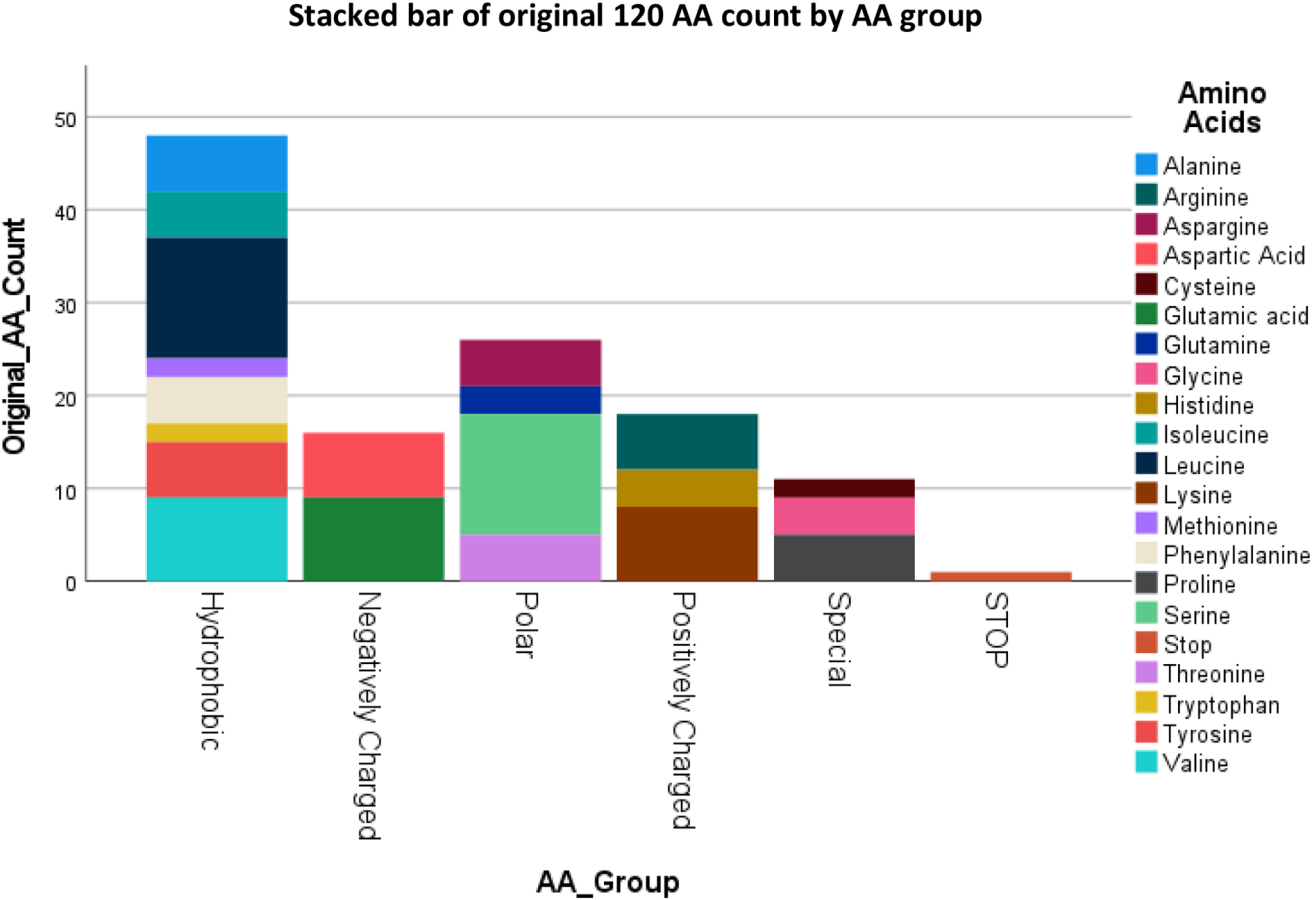
Counts of the original 119 AA of B2M by their Group with the stop codon at the end, result of 120.

**Figure 2** summarizes the individual AA’s and AA groups for the original B2M protein sequence. The B2M gene has 1 stop codon and its transcription will result in Cysteine, Methionine and Tryptophan having only 2 residues in the final protein, and Glutamine having 3 residues out of the total 119 AA’s. The Hydrophobic AA’s are more frequently present compared to AA’s belonging to other chemical groups. The percentages represent the number of AA’s in each group.

### 3.3 SNP AA and AA group change

**Figure 3:**
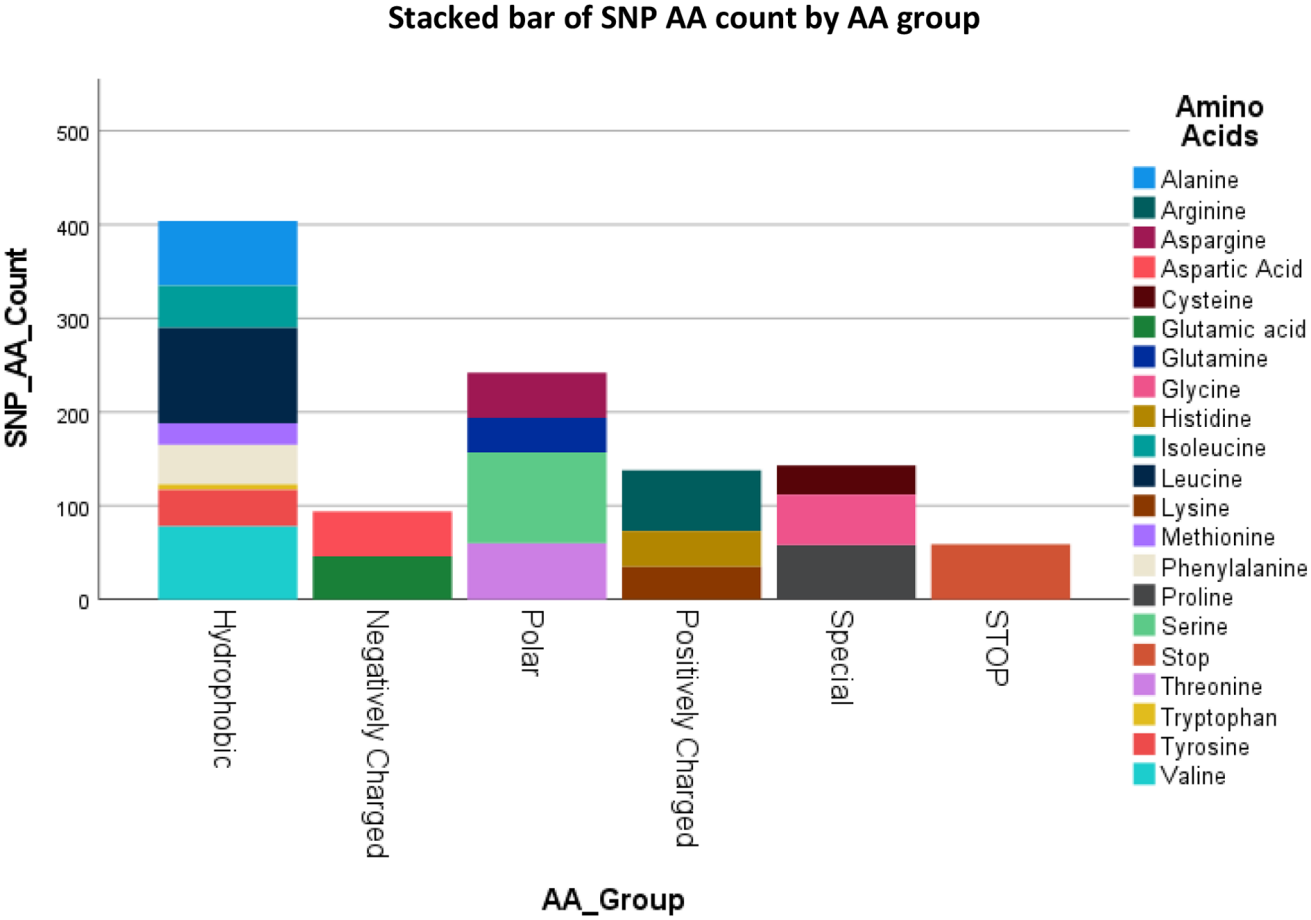
Counts of the occurrence of SNP’s Amino Acids’s and Amino Acids groups of B2M protein. Total results are 1080 (360 nucleotides X 3 SNP’s each = 119 original AA and 1 Stop Codon X 9 SNP’s each).

**Figure 4:**
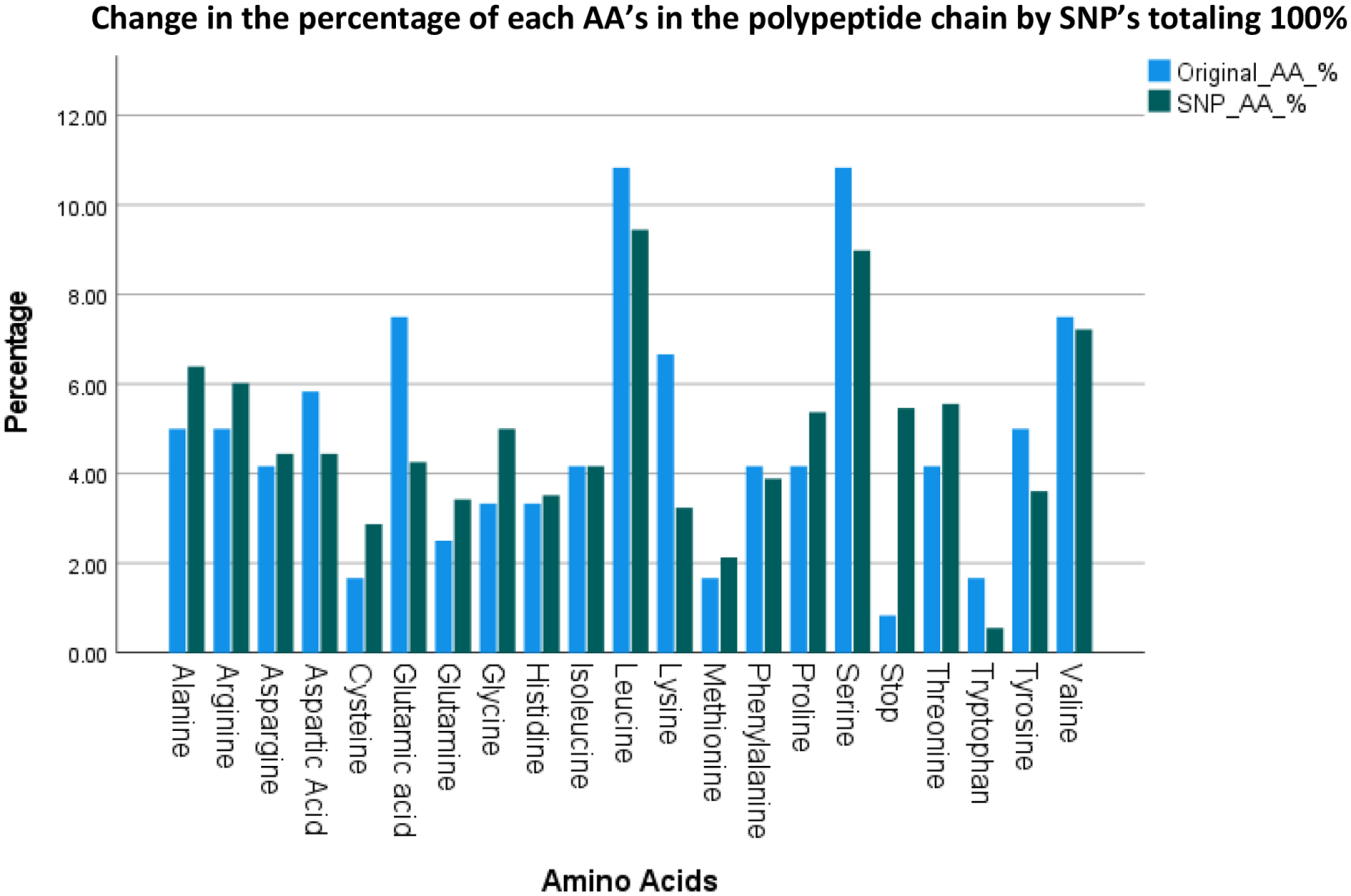
Changes in the frequency of occurrence of each AA between Original Protein (for each AA out of 120) and the SNP process (for each AA out of 1080 SNP’s).

**Figure 5:**
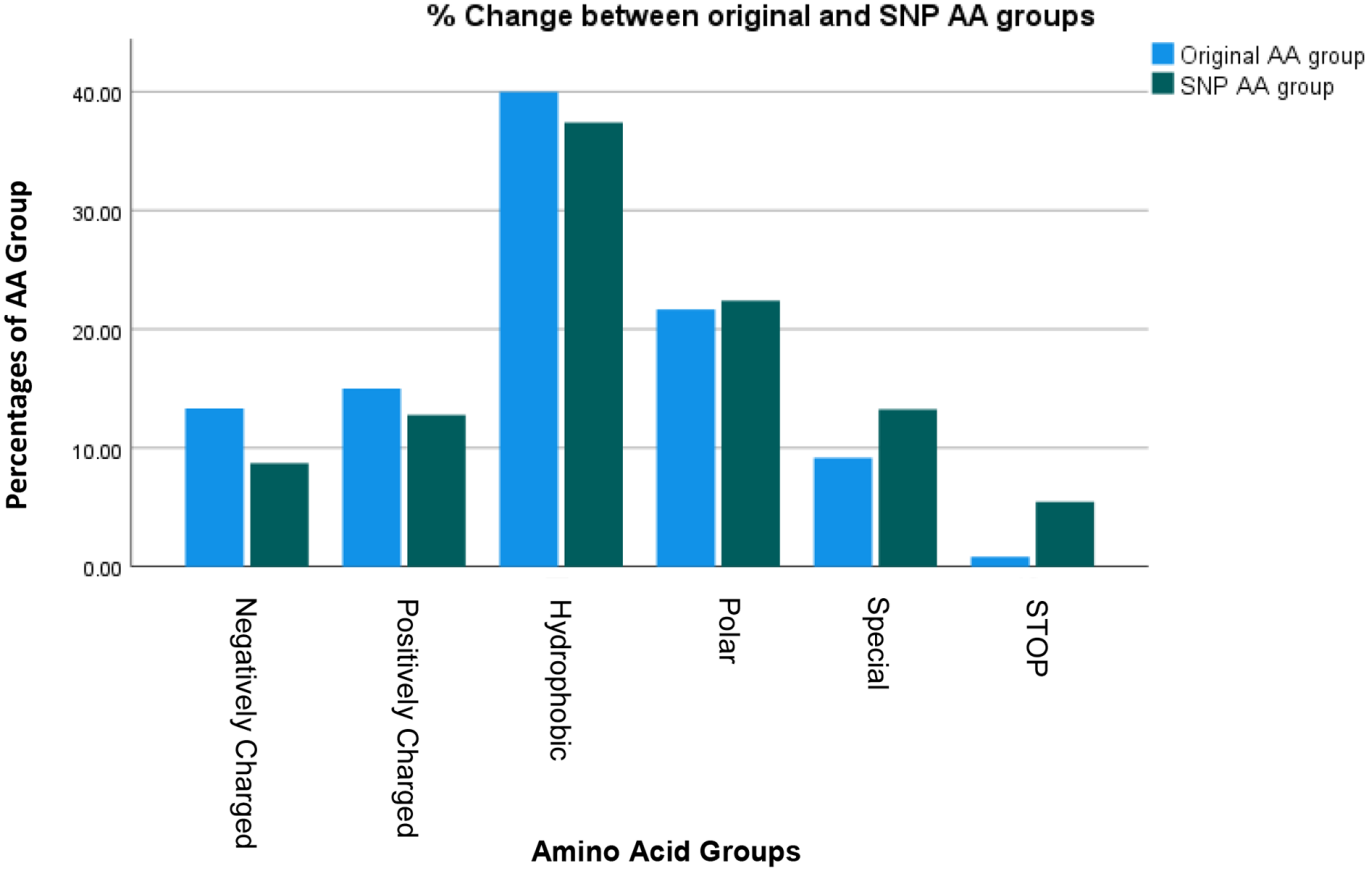
Changes in the frequency of occurrence of each AA Group between Original Protein and the SNP process.

**Figure 3** lists the resulting AA’s and their groups by changes due to the polymorphisms totaling 1080 possible SNP’s. **Figure 4** summarizes the changes in the frequency of each AA and **Figure 5** summarizes the changes for the AA groups. Out of 1080 SNP’s, 239/1080 (22.1%) of SNP’s did not lead to AA substitutions, 274/1080 (25.4%) were substituted by a similar AA (same group of AA), and in 57/1080 (5.3%) the SNP resulted in a STOP codon being the most striking change. In 510/1080 (47.2%) SNP’s, the substituted AA was of a different group and this change had a potentially large effect. The largest changes in the frequency for an AA was for Glutamic Acid and Tryptophan with a decrease from around 7 to 4% and 1.8 to 0.5% respectively, but in most of the AA and Groups almost similar rates of occurrence were observed. (**Appendix 1** shows the total EXCEL sheet with original AA and SNP-induced nucleotide change, AA, AA group and Sneath Factor of Change for the AA).

### 3.4 Effects of SNP on special AAs: the case of methionine and cysteine and stop codons

**Table 1** summarizes the resultant changes for special AA’s and the Stop codon. For Methionine (a hydrophobic essential AA at the start and appearing before the Stop Codon, is encoded by only 1 triplet), SNP’s-induced substitutions generated 12 Hydrophobic AAs, 4 Positively charged and 2 Polar AAs. For Cysteine (a Special group of AA encoded by 2 possible triplets only), SNP-induced substitution generated 6 Hydrophobic, 2 Positively Charged, 4 Polar, 2 similar AA and 2 Stop Codons. As for the Stop Codon (coded by any of 3 triplets), SNP-induced substitutions generated 2 Stop Codons, 3 Hydrophobic, 2 Polar and one positively and negatively charged AA. These substitutions will elongate the polypeptide chain beyond the original sequence until another Stop codon is encountered. In **Appendix 2**, a table with all SNP-induced substitutions for all AA’s (total 1080 SNP’s) is attached.

**Table 1:** List of the resulting SNP’s substitutions for special cases of AA and Stop codon.

Methionine (18 SNP's)	Cysteine (18 SNP's)	Stop Codon (9 SNP's)
Isoleucine 6	Stop Codon 2	Stop Codon 2
Lysine 2	Serine 4	Tyrosine 2
Leucine 4	Phenylalanine 2	Glutamic Acid 1
Arginine 2	Glycine 2	Lysine 1
Threonine 2	Arginine 2	Leucine 1
Valine 2	Tryptophan 2	Glutamine 1
	Tyrosine 2	Serine 1
	Cysteine 2	

Methionine and Cysteine Codons occurred 2 times each and a single Stop Codon at the end. **Appendix 2** gives the tabulation of all SNP-induced substitution of each AA.

### 3.5 Sneath factor of chemical resemblance of AA’s substitutions by SNP’s

**Figure 6:**
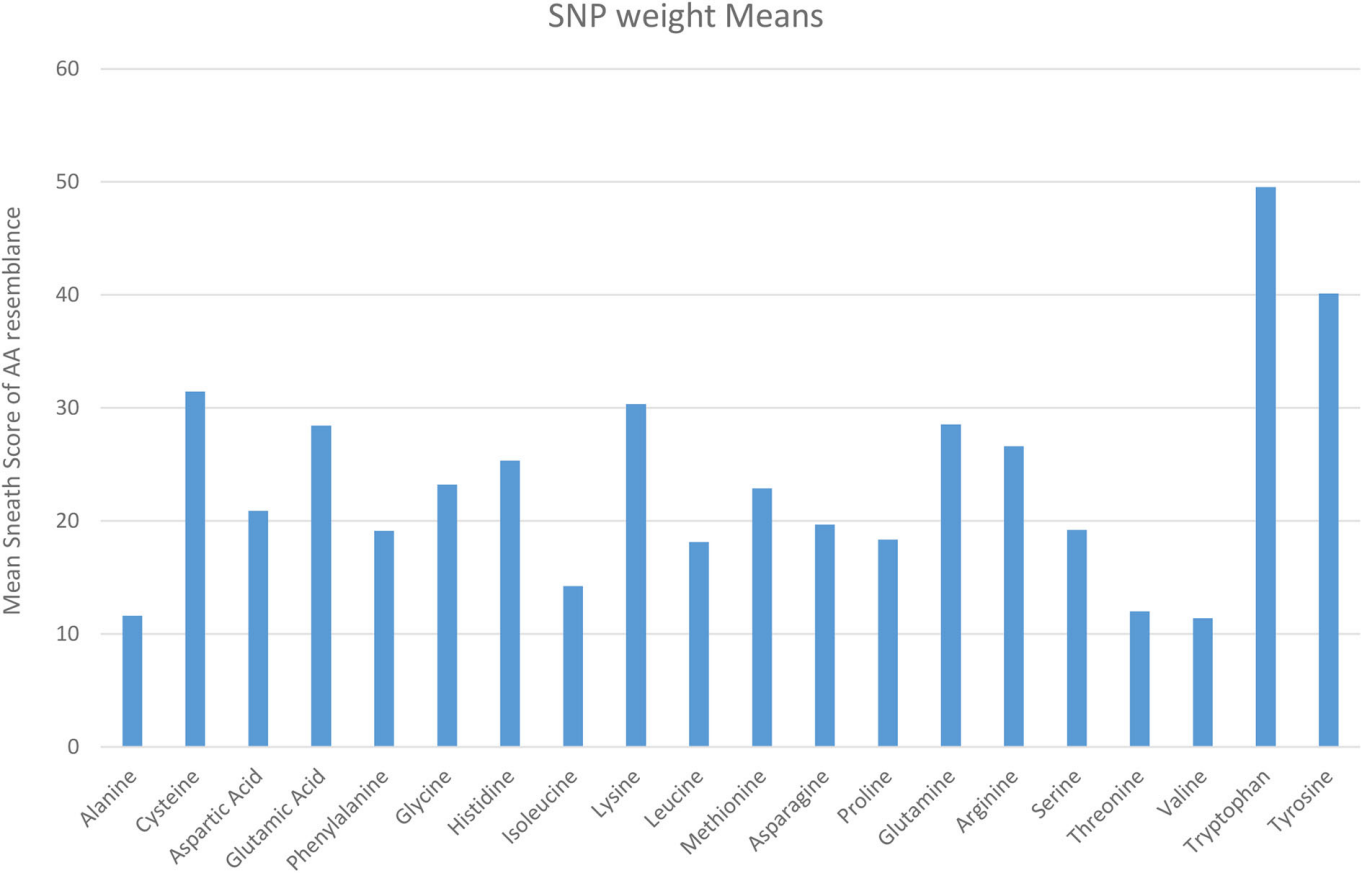
Average sneath factor of chemical resemblance for the original AA by the type of SNPs AA Substitutions (Sneath Factor between 5-43, 0 used if No Change in AA and 100 if a Stop Codon is found).

**Figure 6** lists the average weight of the Sneath Factor for each AA substitution. A factor of 0 represents no change of AA and 43 as the largest change. A score of 100 was chosen to represent the nonsense mutation generating a Stop Codon. The highest scores were SNP-induced substitutions to Tyrosine and Tryptophan because of the higher molecular weights, their Sneath Factor values and likelihood of generating Stop codons (Tyrosine 12/54 and Tryptophan 4/18). The next highest score was observed for Cysteine due to the frequency of SNPs generating stop codons. It is clear that not all substitutions of all AA are possible by the SNP processes.

## 4 Discussion

Currently the amount of bioinformatics tools available freely is huge and attempts to better understand the association of the Genotype to the Phenotype are needed. The use of computer power in dealing with BigData is feasible, but needs better computer modeling, starting from the DNA sequence till the disease phenotype seen at the end. This trial of modelling on Primary Protein structure is the easiest as the rules of coding for AA’s are straight forward. However, better mathematical modelling for Secondary, Tertiary and Quaternary structures are to be discovered and reached.

The Immunoglobulin Domain Gene Superfamily members are widely found in nature with many example of molecules involved in receptor functions and recognition of ligands. They are the cornerstone for the adaptive immune functions, both humoral or cellular and even innate immune responses. B2M was chosen because it is the smallest one, made of one polypeptide chain and a single domain which is highly conserved in nature among all vertebrate animals. What are the mechanisms of preventing SNP’s from changing this protein and how they work differently from other proteins? Is there a critical role for and application of the disulfide bond between far apart Cysteine molecules during translation? These questions need to be better studied (24).

This study aimed to mathematically model the theoretical effects of the SNP’s on the protein’s Primary structure. In 47.5% of times, SNP’s in the B2M gene led to no or minimal effect, while in 52.5% of times, a catastrophic or major effect is expected. There were only 2 previous SNP’s of B2M associated with disease as mentioned in the literature search in the introduction segment.

Previous work that compared the presence of SNP’s in B2M to 3 other proteins (Cystatin C, Retinol Binding Protein and Transthyretin) found that no SNP’s were seen in 500 normal individuals with longitudinal follow-up for modifications. Only in 2.4% of the sample for B2M with 6 months follow-up, there was a posttranslational removal of Lys 58 (des-K 58). This phenomenon is thought to result in an effect on C1s (a Complement Component 1 part with C1q and C1r). The other proteins in that study (Cystatin C, Retinol Binding Protein and Transthyretin) had 7, 5 and 41 SNP modifications in the sample of 500 subjects respectively (25).

Obvious limitations in this study are multiple. We need to take into account the SNP’s effects on the Secondary, Tertiary and Quaternary structures of the protein and their interactions with the Primary structure changes like the important disulfide bond that maintains the globular structure of this protein.

This study evaluated only the coding mature part of the B2M gene and there is need to further study the effects of SNP’s in coding and noncoding segments of the gene. SNP’s might interact with other processes like the DNA repair mechanisms, post transcriptional and post translational modifications.

Chemical nature of the AA is significant but alone cannot explain all of the effects seen because of the substitutions. In the 2 clinically reported cases of B2M SNP’s of Hereditary Systemic Amyloidosis Type 6 (the change carries Sneath Factor of Resemblance of only 14 of the possible range of 5-43) and Familial Hypercatabolic Hypo-proteinemia with Immunodeficiency (with the Sneath Factor of Resemblance change was only 16) for the AA substitutions seen. With more genetic data available and more powerful computing powers, better methods of evaluating the effects of AA substitutions have to be agreed upon better than just the mere chemical resemblance between AA pair of Sneath.

An alternative approach to studying the issues at hand is to use computing power of Big Data and available whole genome or exome Sequences. The challenge is to match within these databanks between the phenotypes and genotypes in search of correlations for specific SNP’s or other mutations and diseases. Supervised Machine Learning and Artificial Intelligence are candidate tools to utilize. This process of genotype/phenotype correlation has to be repeated every now and then with more data as new associations might be found.

Another approach is experimental, by studying the SNP effect on a protein. We have to start using genetic engineering tools like CRISPR-Cas9 not only for therapeutic but also for research purposes such as inducing SNP’s at selected places like the Cysteine residues of the B2M gene in an appropriate model of Antibody molecule like the monoclonal antibody hybridomas and observe their effects. Animal models of Inbred Strains of Mice might be another alternative to cause SNP’s and observe their effects.

## 5 Conclusions

1. The SNP process is an important “random” phenomenon that can modify variably the Primary structure then function of proteins from no or minimal change in almost half of the cases to major or catastrophic changes in the rest as estimated from the example of B2M.
2. For conserved molecules in nature such as the Immunoglobulin Domain like B2M, there has to be mechanisms of control against modifications in the DNA sequences or SNP’s occurring and better understanding of these control mechanisms is needed.
3. Better mathematical and computer modeling is needed to study Genetics, its’ processes of transcription, translation and all levels of Protein Structure leading to phenotypes and disease associations. Our study achieved this goal for the effects of SNP’s on the Primary structure of a protein. Artificial Intelligence and Supervised Machine Learning are suitable candidates to deal with the Big Data available.
4. Further studies are needed to experiment with the SNP’s impact on proteins of interest and observe their structural and functional effects by using genetic engineering tools in appropriate cell lines, hybridomas or inbred strains of mice.

## Data Availability

The original contributions presented in the study are included in the article/**Supplementary Material**. Further inquiries can be directed to the corresponding author.
